# A Bibliometric and Critical Review of Cellulose-Based Aerogels for Wastewater Treatment

**DOI:** 10.3390/gels12070643

**Published:** 2026-07-18

**Authors:** Fengyun Sun, Mingqiao Wang, Shizuo A. Niu, Xiaodong Zhu, Kefa Ren, Yingge Zhang, Yaru Yang, Dong Liu

**Affiliations:** 1 School of Mechanical Engineering, Chengdu University, Chengdu 610106, China; sfy026@163.com (F.S.); 18582865702@163.com (M.W.); 202510116427@cdu.edu.cn (S.A.N.); xiaodangjia21@126.com (X.Z.); 2 College of Earth and Planetary Sciences, Chengdu University of Technology; Chengdu 610059, China; xyueren@163.com; 3 Sichuan Tuomai Xingzhong Technology Co., Ltd., Chengdu 610106, China

**Keywords:** cellulose aerogel, advanced wastewater treatment, bibliometric analysis, adsorption-photocatalysis synergy, research frontier

## Abstract

The treatment of refractory wastewater pollutants requires advanced materials capable of synergistic enrichment and destruction. Cellulose-based aerogels, combining biomass sustainability with a porous structure, are a promising platform, yet a quantitative synthesis of this field’s evolution is lacking. This study presents the first bibliometric and visual analysis of 463 publications on cellulose-based aerogels for wastewater treatment. The field shows S-shaped growth, evolving from a Nascent phase to Exponential Growth and now entering Maturation. Social network analysis reveals a China-centered but increasingly international collaboration pattern, while institutional productivity remains fragmented into multiple small research teams. The foundation rests on two synergistic pillars: adsorptive sequestration and catalytic degradation, with research evolving from their parallel development to active fusion. Current frontiers focus on sustainable system engineering, emphasizing process integration, material regeneration, and advanced precursors like cellulose nanofiber. This analysis maps the field’s maturation from material exploration toward integrated catalytic system design, providing a foundational reference and clear directives for future research to address integration challenges. In addition to bibliometric mapping, this review critically discusses treatment functions (adsorption, catalytic oxidation, and integrated pathways) and deployment barriers (regeneration, recyclability, and scale-up feasibility), thereby linking knowledge evolution to practical wastewater-treatment translation. While China contributes the largest publication share in the present dataset, the field is supported by increasing participation from multiple countries and regions.

## 1. Introduction

Treating wastewater that contains refractory pollutants—especially synthetic dyes, toxic metal ions, and other persistent contaminants—remains a global environmental priority because these species are difficult to remove and can cause long-term ecological and health damage [1,2,3,4,5]. Conventional standalone treatments (e.g., biological and physical routes) often fail to achieve deep purification or complete detoxification, particularly for complex mixed-pollutant matrices [6,7,8]. For this reason, advanced oxidation processes (AOPs), including photocatalytic and photo-assisted catalytic systems, have been widely investigated due to their ability to generate highly reactive species for pollutant breakdown [9,10,11,12,13,14]. Even so, practical deployment is still limited by challenges such as catalyst recovery, reuse stability, regeneration durability, and process economics [11,15,16].

Within this context, cellulose-based aerogels have become attractive candidates for water remediation. Their appeal comes from the combination of renewable biomass origin and favorable 3D porous architectures, such as high specific surface area, interconnected pore channels, and tunable surface chemistry [1,2,4,17,18]. In addition to direct adsorption, these aerogels can serve as multifunctional scaffolds for catalytic components, enabling coupled “capture-and-degrade” treatment pathways [19,20,21,22]. This coupling is functionally important: adsorption enriches contaminants near active interfaces, while catalytic reactions subsequently promote in situ degradation, improving both overall removal efficiency and active-site utilization [9].

**Figure 1:**
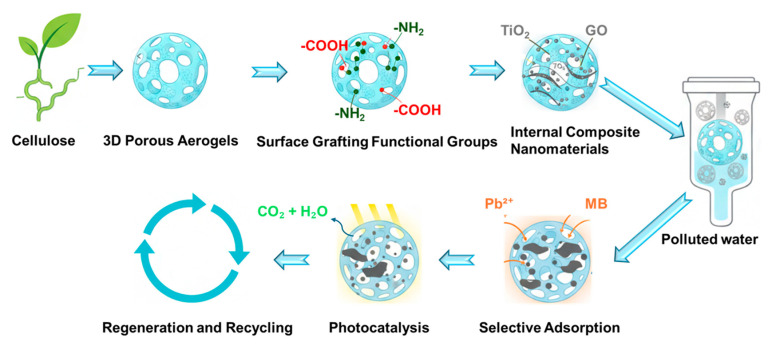
Schematic illustration of the circular design of cellulose-based aerogels for water purification via synergistic adsorption and photocatalysis. Colors are used only for visual distinction and do not represent quantitative data categories.

As shown in Figure 1, the technical logic of cellulose-aerogel-based purification can be described as a circular pathway covering renewable cellulose sourcing, aerogel construction, targeted functionalization, contaminant capture/degradation, and material regeneration/reuse [23,24,25]. Such a process-level perspective is consistent with the current transition from single-function sorbents toward integrated and potentially scalable treatment platforms.

Existing review literature has already established important foundations for this field. Mautner reviewed nanocellulose membranes and filters for water treatment, emphasizing the importance of surface charge, porosity, and functional modification in filtration and contaminant removal [26]. Salama et al. provided a broad review of nanocellulose-based materials for water treatment, covering adsorption, photocatalytic degradation, disinfection, antifouling, and nanofiltration [27]. From the perspective of porous cellulose materials, Lavoine and Bergström summarized the processing, properties, and applications of nanocellulose-based foams and aerogels [28], while De France et al. reviewed hydrogels and aerogels containing nanocellulose from a materials-design perspective [29]. In addition, Hokkanen et al. discussed modification strategies for cellulose-based adsorbents to improve adsorption performance [30]. These studies provide valuable material-level and mechanism-level insights into cellulose-based water-treatment materials, especially regarding fabrication routes, surface functionalization, adsorption enhancement, and selected application scenarios.

Despite these contributions, the existing literature remains dominated by narrative reviews and material-centered discussions. Most prior reviews summarize synthesis protocols, surface modification strategies, compositional design, adsorption mechanisms, or selected performance indicators such as adsorption capacity, kinetics, and cycling behavior. However, they generally do not provide a quantitative field-level map showing how the research landscape has evolved over time. In particular, systematic evidence is still needed to clarify publication trajectories, collaboration networks, intellectual bases, co-citation clusters, keyword bursts, and temporal shifts in research topics. This distinction is important because cellulose-based aerogels are no longer limited to single-function adsorption systems; they are increasingly being developed as integrated platforms that combine contaminant capture, catalytic or photocatalytic degradation, regeneration, reuse, and process-oriented design.

To address this gap, this study performs a bibliometric and science-mapping analysis of cellulose-based aerogel research for wastewater treatment, based on 463 publications indexed in the Web of Science Core Collection from 2011 to 2025. Using CiteSpace, we analyze publication trajectories, country and institutional collaboration networks, co-citation structures, keyword clusters, burst terms, and thematic evolution to identify core knowledge domains and emerging research fronts [31,32,33,34]. Compared with conventional narrative reviews that primarily summarize material recipes and single-point removal performance, this study establishes a bibliometric-to-engineering bridge by integrating knowledge-structure mapping with a function-oriented critical discussion of wastewater-treatment applications.

The novelty of this review therefore lies in three aspects. First, it provides a reproducible bibliometric overview of cellulose-based aerogels for wastewater treatment over a 15-year period, rather than summarizing selected experimental studies. Second, it links quantitative science mapping with practical treatment functions, including adsorption, catalytic/photocatalytic degradation, coupled capture-and-degrade processes, regeneration, and reuse. Third, it interprets the evolution of this field from the perspective of engineering translation, with particular attention to operational stability, recyclability, scalability, and compatibility with realistic wastewater matrices. Accordingly, this review has two linked aims: (i) to quantitatively characterize the bibliometric landscape of cellulose-based aerogels for wastewater treatment, and (ii) to critically interpret the ongoing shift from single-function removal toward integrated, regenerable, and process-compatible treatment systems.

The remainder of this paper is organized as follows. Section 2 introduces the data source, retrieval strategy, and bibliometric workflow. Section 3 presents the publication landscape and knowledge structure. Section 4 discusses wastewater-treatment functions, adsorption-catalysis integration, and translation-relevant constraints, including regeneration, reuse, and scalability. Section 5 summarizes key findings, limitations, and future perspectives.

It should be emphasized that the scope of this review is deliberately limited to cellulose-based aerogels used in wastewater treatment, water purification, and water-remediation contexts. This study does not aim to evaluate the entire cellulose-based aerogel literature, nor does it draw field-level conclusions for other application domains, such as thermal insulation, packaging, biomedical scaffolds, energy storage, sensors, oil absorption without an explicit water-remediation context, or non-aqueous catalysis. Therefore, terms such as “the field”, “research hotspots”, “emerging frontiers”, and “thematic evolution” in this paper refer specifically to the wastewater-treatment-related corpus retrieved and analyzed in this study, unless otherwise stated.

## 2. Data Collection, Search Strategy, and Bibliometric Workflow

**Figure 2:**
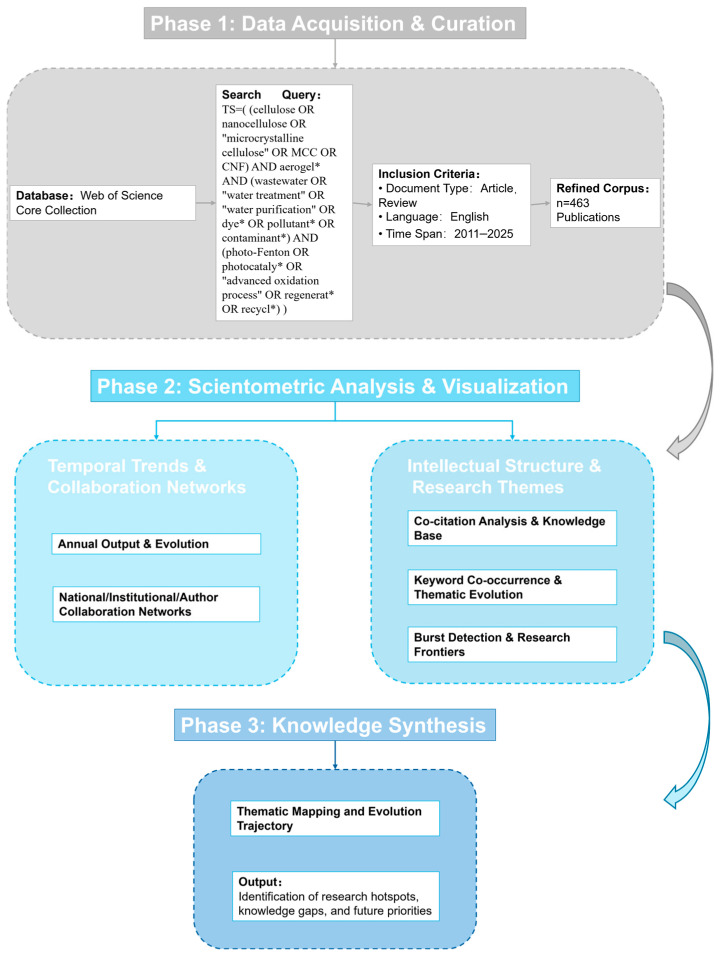
Methodological framework for the bibliometric analysis. An asterisk (*) indicates a wildcard for truncation search.

The overall research workflow for this bibliometric study comprised three sequential phases—data collection and curation, scientometric analysis and visualization, and knowledge synthesis—as schematically illustrated in Figure 2.

### 2.1. Data Source Selection and Study Boundary

The bibliometric dataset was retrieved from the Web of Science Core Collection (WoSCC) on 14 January 2026. WoSCC was selected as the data source for this bibliometric and science-mapping analysis because it provides curated and relatively standardized bibliographic metadata, including titles, abstracts, author information, institutional affiliations, keywords, subject categories, cited references, and citation records. These metadata are essential for reproducible co-authorship, co-citation, keyword co-occurrence, and burst-detection analyses. In addition, WoSCC offers comparatively consistent cited-reference formatting and citation indexing, which is particularly important for CiteSpace-based network construction, clustering, and temporal comparison across time slices. Using a single curated citation database also helps reduce duplicate records and metadata heterogeneity that may occur when combining multiple databases with different indexing policies, document coverage, and reference formats.

This choice was made for methodological consistency and reproducibility rather than to imply that WoSCC covers all relevant literature. The use of WoSCC inevitably introduces database-coverage limitations. Relevant studies indexed only in other sources, such as Scopus, PubMed, Google Scholar, Engineering Village, CNKI, patents, conference proceedings, dissertations, technical reports, or non-English literature, may not be fully represented in the present corpus. Therefore, the bibliometric results reported in this study should be interpreted as patterns within the predefined WoSCC-based wastewater-treatment corpus, rather than as exhaustive measurements of the entire global literature on cellulose-based aerogels.

The retrieval strategy was designed to identify studies on cellulose-based aerogels in wastewater-treatment or water-purification contexts. The Boolean query combined four mandatory concept groups: cellulose-related terms, aerogel-related terms, wastewater/water-purification terms, and treatment/regeneration-related terms. The exact query was as follows: TS = ((cellulose OR nanocellulose OR “microcrystalline cellulose” OR MCC OR CNF) AND aerogel* AND (wastewater OR “water treatment” OR “water purification” OR dye* OR pollutant* OR contaminant*) AND (photo-Fenton OR photocataly* OR “advanced oxidation process” OR regenerat* OR recycl*)).

The search terms were selected to balance sensitivity and specificity within the predefined wastewater-treatment scope. The first concept group, including cellulose, nanocellulose, microcrystalline cellulose, MCC, and CNF, was used to capture cellulose-derived aerogel matrices and common cellulose-based building blocks. The broad term “cellulose” was retained because many studies describe materials using phrases such as cellulose nanofibers, cellulose nanocrystals, bacterial cellulose, regenerated cellulose, or modified cellulose without using a single standardized abbreviation. Highly ambiguous abbreviations were not used alone unless they were expected to be disambiguated by the co-occurrence of aerogel- and wastewater-related terms, in order to reduce false-positive retrieval. The second concept group, aerogel*, restricted the corpus to aerogel-type porous materials. The third group, including wastewater, water treatment, water purification, dye*, pollutant*, and contaminant*, was designed to identify water-remediation contexts, because many relevant studies report model pollutants such as dyes, heavy metals, or organic contaminants without explicitly using the term “wastewater” in the title or abstract. The fourth group, including photo-Fenton, photocataly*, advanced oxidation process, regenerat*, and recycl*, was used to emphasize advanced treatment functions, catalytic/oxidative pathways, and reusability, which are central to the practical deployment of cellulose-based aerogels in wastewater treatment. Overall, the query was intended to retrieve a focused and reproducible corpus of wastewater-treatment-related studies rather than the entire body of cellulose aerogel literature.

The initial retrieval yielded 520 records. After applying the predefined time-span, language, document-type, deduplication, and title/abstract screening criteria, 463 records were retained as the final bibliometric corpus. All records were exported from WoSCC as full records with cited references and were used for subsequent bibliometric analyses, including collaboration network analysis, co-citation analysis, keyword analysis, and burst detection. The complete query string, stepwise screening workflow, inclusion and exclusion criteria, adsorption-oriented validation check, and full list of the 463 included publications are provided in Supplementary Files S1 and S2.

### 2.2. Data Screening and Curation

The retrieved records were screened according to predefined inclusion and exclusion criteria. Eligible records were required to meet three conditions: (i) they involved cellulose, nanocellulose, microcrystalline cellulose, or cellulose-derived components; (ii) they focused on aerogel-based materials; and (iii) they were related to wastewater treatment, water purification, pollutant removal, catalytic/photocatalytic degradation, regeneration, recycling, or other relevant water-remediation functions. Records were excluded when they were outside the wastewater/water-treatment context, did not involve aerogel materials, or were not substantively related to cellulose-based systems. Title and abstract screening was used to confirm eligibility after the application of WoSCC filters and deduplication. Studies on cellulose-based aerogels for thermal insulation, packaging, biomedical applications, energy storage, sensors, oil absorption without an explicit wastewater or water-remediation context, or other non-water-treatment applications were excluded from the quantitative corpus and were not used to support field-level conclusions in this review.

Because the main query was designed to capture multiple wastewater-treatment functions rather than adsorption alone, an adsorption-oriented validation check was conducted after finalizing the corpus. Within the 463-record corpus, records containing adsorption-related descriptors, including adsorp*, adsorbent*, sorp*, and sequestrat*, were identified at both topic and title levels. The validation check identified 342 topic-level and 140 title-level adsorption-related records within the predefined corpus, as well as five additional high-specificity title-level records outside the corpus. These additional records were considered in the qualitative discussion, whereas the quantitative bibliometric analyses remained based on the predefined 463-record corpus. The detailed validation queries, stepwise filtering log, and validation records are provided in Supplementary File S1.

### 2.3. Scientometric Tools and Parameter Settings

The scientometric analyses were conducted using CiteSpace (version 6.4.R1), following the Phase 2 framework in Figure 2. The analytical workflow encompassed co-authorship network analysis (country, institution, author levels) to map collaboration, document co-citation analysis to trace the intellectual base, keyword co-occurrence analysis to identify research themes, and keyword burst detection to pinpoint emerging frontiers.

### 2.4. Workflow of the Bibliometric Analysis

In CiteSpace, the time slicing was set to one year from 2011 to 2025. Networks were constructed using the g-index (k = 25; Top N = 50 per slice) and pruned with the Pathfinder algorithm to improve network readability. Other parameters remained at their default settings. In all CiteSpace network visualizations in this study, node size represents the frequency or output of the analyzed item, depending on the network type; links indicate collaboration, co-occurrence, or co-citation relationships; and node colors correspond to temporal slices according to the year color scale shown in each figure. Cooler colors denote earlier years, whereas warmer colors denote more recent years. Purple outer rings, when present, indicate nodes with relatively high betweenness centrality. Insights from these analyses were synthesized, as summarized in Figure 2, to identify hotspots, gaps, and future priorities.

## 3. Bibliometric Landscape and Knowledge Structure

### 3.1. Publication Growth and Research Phases

**Figure 3:**
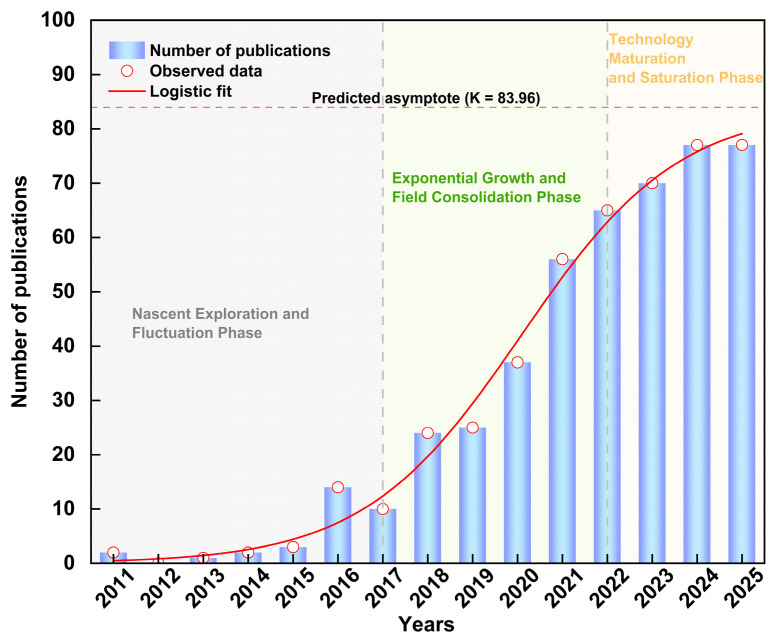
Annual publication output (2011–2025) of cellulose-based aerogel research for wastewater treatment. Colors and shaded regions correspond to the categories and developmental phases indicated in the legend and in-figure labels.

The annual publication output (2011–2025) reveals a distinct S-shaped growth trajectory when fitted with a three-parameter logistic function (Slogistic1 model, R^2^ = 0.990), indicating a clear transition from emergence to maturation (Figure 3). The logistic fitting yielded a model-predicted asymptote of K = 83.96 publications per year for the WoSCC-defined wastewater-treatment dataset, which is shown as a dashed horizontal line in Figure 3. Because this asymptotic value depends on the selected database, search strategy, time window, and fitting model, it should be interpreted as an indicative bibliometric parameter rather than as a deterministic prediction of future publication output. Based on the model’s inflection point (2020.1), the field’s development is segmented into three phases.

The initial Nascent Exploration Phase (2011–2017) was characterized by low and fluctuating annual outputs, reflecting foundational, proof-of-concept research. This was followed by a period of Rapid Growth and Consolidation (2018–2022), centered on the inflection point, during which annual publications increased sharply, signaling the field’s acceleration and mainstream acceptance. Since 2023, the field has entered a Maturation Phase, in which the annual publication output approaches the model-predicted asymptote of approximately 84 publications per year, suggesting a strategic shift from expansive growth toward deeper, application-focused research within the established paradigm. This evolutionary pattern provides a quantitative context for analyzing the subsequent thematic and intellectual shifts within the field.

The increase in publication output is closely associated with the evolution of cellulose-aerogel synthesis technologies rather than with a purely bibliometric expansion. During the Nascent Exploration Phase, most studies relied on relatively simple sol–gel processing, freeze-drying, physical blending, and basic chemical crosslinking to construct lightweight cellulose or nanocellulose aerogels. These early systems demonstrated the feasibility of using renewable, porous, and surface-functional cellulose networks for pollutant capture, but their synthesis routes were often limited by weak wet stability, insufficient mechanical robustness, limited control over pore architecture, and relatively simple adsorption-dominated functions. These technical constraints help explain the low and fluctuating publication output before 2018.

The rapid increase after 2018 coincided with several synthesis advances that expanded both the design space and the wastewater-treatment functions of cellulose-based aerogels. Directional freezing, ice-templating, covalent/ionic dual crosslinking, and hierarchical pore engineering improved mass transfer, shape stability, and mechanical integrity. At the same time, interfacial assembly and in situ growth strategies enabled the incorporation of metal oxides, carbonaceous components, magnetic particles, MOF-derived structures, and photocatalytic or photo-Fenton active phases into cellulose networks. These advances transformed cellulose aerogels from mainly passive adsorbents into multifunctional platforms capable of adsorption, catalytic/photocatalytic degradation, oil/water separation, regeneration, and reuse. Therefore, the inflection period around 2020 can be interpreted as a point at which synthesis methods became sufficiently versatile to support broader functional integration and application-oriented studies. Since 2023, the slower growth approaching the model-predicted asymptote suggests not a decline in relevance, but a transition from method expansion toward technology maturation, with increasing attention to scalable fabrication, structural durability, cyclic stability, and performance in complex water matrices [35,36,37].

### 3.2. Analysis of Research Power and Collaboration Networks

This section analyzes the global distribution of research output and collaborative patterns to delineate the field’s social architecture, contextualizing their evolution within the three-phase developmental trajectory established in Section 3.1. Beyond mapping productivity, the collaboration network is interpreted here as an innovation infrastructure that shapes how synthesis strategies, functionalization methods, pollutant-removal mechanisms, and application-oriented validation practices diffuse and recombine across countries, institutions, and research teams.

#### 3.2.1. Global Research Output and National Collaboration Network

**Figure 4:**
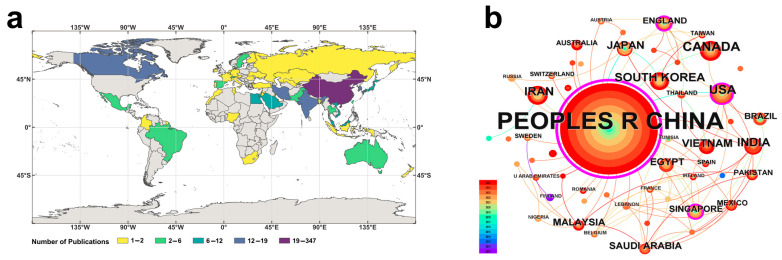
Global research landscape of cellulose-based aerogels for water purification. (**a**) Geographic distribution of publication output. (**b**) Network of international co-authorship.

The global research landscape is characterized by extreme geographical concentration, with East Asia serving as the undisputed epicenter of research productivity. As illustrated in Figure 4a and quantified in Table 1, this concentration is predominantly anchored by the People’s Republic of China, which is the overwhelmingly dominant contributor, accounting for 347 publications (75% of the analysed publications). Significant but substantially lower output originates from North America and Europe, with sporadic contributions from regions in the Middle East and Southeast Asia. China’s early entry (2014) and its exceptionally high network centrality (1.07) have firmly established it as the foundational hub and primary driver of the field’s global activity [38,39].

The international co-authorship network (Figure 4b, N = 53, E = 96) exhibits a distinct star-shaped topology centered on China. This structure indicates that China serves as the primary knowledge producer and the principal conduit for global collaboration. In contrast, other productive nations (e.g., Canada, USA, Iran, India) exhibit low to moderate centrality, suggesting their collaborative roles are predominantly bilateral with the central hub rather than forming dense, multilateral sub-networks.

This hub-and-spoke architecture aligns with the field’s phased growth. During the Nascent Phase, China’s early start provided the initial knowledge base. The subsequent entry of other nations (e.g., Iran, India, Vietnam) during the Exponential Growth Phase often occurred through collaboration with Chinese groups, facilitated by this central hub model to accelerate paradigm consolidation. In the current Maturation Phase, while the research paradigm is globally shared, the network remains sparse outside the central hub, indicating that deep multilateral collaborations among non-hub nations are still nascent.

International collaboration and innovation diffusion—the topology of the national co-authorship network indicates that international collaboration influences innovation through two contrasting mechanisms. On the positive side, the China-centered hub-and-spoke structure facilitates rapid outward diffusion of dominant technical paradigms, including cellulose-aerogel synthesis, surface functionalization, adsorption mechanisms, and adsorption–catalysis integration. This hub-mediated diffusion likely helped later-entering countries participate quickly in the field and contributed to the rapid consolidation of research themes after 2018. On the other hand, innovation in cellulose-based aerogels for wastewater treatment increasingly requires the recombination of heterogeneous expertise, such as biomass materials chemistry, interfacial engineering, photocatalysis/Fenton chemistry, membrane or reactor design, regeneration testing, and validation in complex water matrices. The sparse links among non-hub countries suggest that opportunities for multidirectional knowledge recombination and independent cross-context validation remain limited. Therefore, the future innovation capacity of the field may depend not only on maintaining strong links with the central hub, but also on developing more distributed multilateral collaborations among non-hub countries and application-oriented teams. Such collaboration would help transform dispersed laboratory advances into more robust, transferable, and deployable wastewater-treatment technologies [40,41,42,43].

#### 3.2.2. Institutional Contribution and Collaboration Network

**Figure 5:**
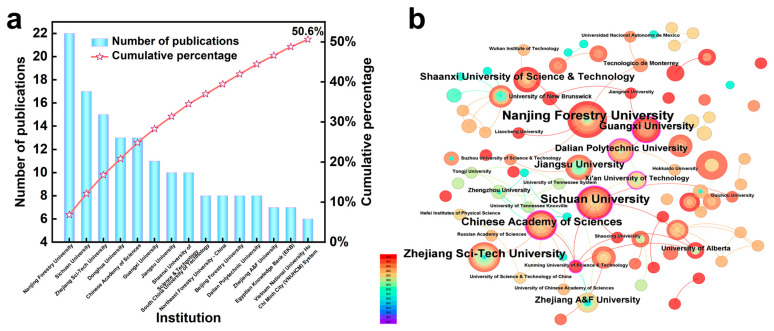
Institutional contribution and connectivity. (**a**) Ranking of leading institutions by publication volume. (**b**) Network of institutional interconnections.

Institutional-level analysis reveals a concentrated yet fragmented landscape that has shaped the field’s development. Productivity, as shown in the Pareto chart (Figure 5a) and Table 2, follows a highly skewed distribution: the top four institutions (Nanjing Forestry University, Sichuan University, Zhejiang Sci-Tech University, and Donghua University) collectively contributed 50.6% of the output from the top 15, indicating a pronounced high concentration.

Temporal analysis of first publication years delineates institutional roles across the field’s growth phases. Early entrants like Zhejiang Sci-Tech University (2016) were active in the Nascent Phase. Current leaders such as Sichuan University (2018) and Nanjing Forestry University (2017) emerged during the Exponential Growth Phase, driving expansion. The recent entry of institutions like Guangxi University (2022) reflects the ongoing geographical diffusion characteristic of the Maturation Phase.

A critical insight emerges from the divergence between publication volume and network influence. While Nanjing Forestry University leads in output, the Chinese Academy of Sciences (CAS) holds the highest centrality (0.17), positioning it as the key connector. Conversely, several prolific institutions (e.g., Donghua University, Jiangsu University) exhibit zero centrality, indicating isolated or internally focused research activities.

This is corroborated by the institutional collaboration network’s structural metrics (N = 86, E = 81, Density = 0.0222) (Figure 5b). The extremely low density and the fact that the largest connected component comprises only 38% of nodes reveal a fragmented “archipelago” structure of small, poorly connected clusters. CAS acts as a rare bridge among a few clusters. This sparse connectivity suggests that concentrated productivity has not translated into a well-integrated collaborative ecosystem, potentially hindering broad knowledge synthesis and innovation.

In summary, the field is propelled by a small set of highly productive institutions within a weakly connected network. Strategic efforts to enhance inter-institutional collaboration, leveraging pivotal hubs like CAS, are essential to overcome existing knowledge silos and foster the cross-disciplinary synergy needed for future advances. At the institutional level, stronger cross-cluster collaboration could accelerate innovation by linking material-fabrication expertise with advanced characterization, catalytic-performance evaluation, regeneration assessment, and process-level wastewater-treatment validation.

#### 3.2.3. Author-Centric Analysis: Productivity, Impact, and Collaborative Structure

**Figure 6:**
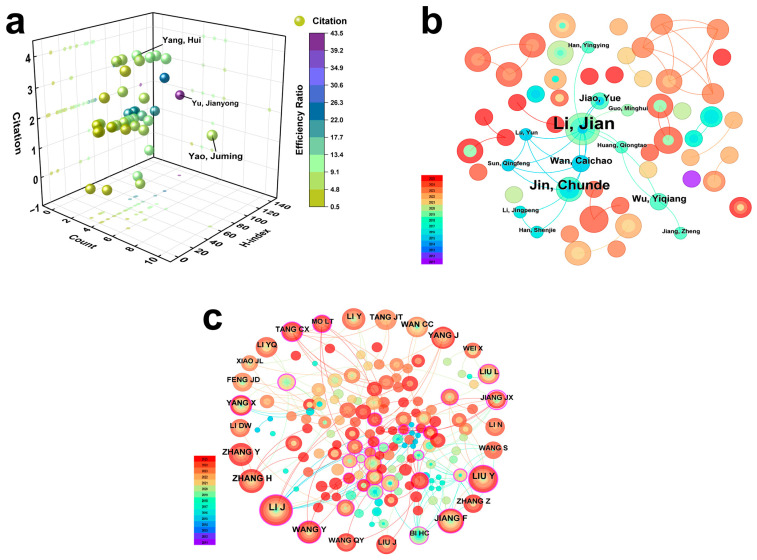
Author-centric analysis of productivity, impact, and collaboration. (**a**) Multidimensional author-level profile based on publication count, WoS author-profile H-index, corpus-level citation frequency, and citation-normalized H-index. (**b**) Macro-level co-authorship network. (**c**) Largest connected component and local team clusters.

An integrated analysis of author productivity, impact, and collaboration networks reveals the micro-dynamics propelling the field. Moving beyond mere publication counts, a multidimensional profile—considering publication volume, WoS author-profile H-index, corpus-level citation frequency, and citation-normalized H-index—delineates distinct contributor patterns, including high-volume producers, authors with strong career-level scholarly profiles, and contributors showing relatively high corpus-level citation visibility (Figure 6a). Data in Table 3 further indicate that productivity, author-profile H-index, and citation frequency within the analyzed corpus are not fully aligned. For example, Gao, Junkuo [44] combines a high WoS H-index with relatively high corpus citation frequency, whereas other productive authors show different balances between career-level influence and topic-specific citation visibility.

The social architecture of collaboration, visualized through co-authorship networks, is characterized by exceptionally low density (Figure 6b,c), corroborating a model of an “archipelago of micro-teams.” Collaborative ties are concentrated within small, isolated clusters, indicating that deep, integrated teamwork is not the norm. A nuanced finding, however, is that 93% of nodes belong to the largest connected component, suggesting field-wide coherence is maintained through indirect pathways and pivotal hub authors (e.g., LI J, LIU Y) [45,46], who act as knowledge brokers across the fragmented landscape.

Cross-referencing productive authors with co-cited authors (Table 3 and Table 4) clarifies distinct pathways to influence. The most productive authors in the corpus are not necessarily the most frequently co-cited intellectual anchors, whereas foundational contributors such as LI J [45], with high centrality and earlier publication years, form part of the cited intellectual backbone. Authors such as Gao, Junkuo [44] illustrate an intermediate pattern by combining relatively high productivity, a strong WoS author-profile H-index, and comparatively high citation visibility within the analyzed corpus.

Collectively, the field appears driven by a dual engine: tightly knit, productive micro-teams that push application frontiers, and a set of influential hub authors and seminal works that provide the cohesive theoretical foundation. This prevalent structure—highly fragmented yet indirectly connected—likely enabled rapid, parallel innovation during the field’s growth phase. As the field matures and faces more complex, interdisciplinary challenges, this same structure may become a limiting factor. Future progress, therefore, may depend on strategic efforts to build collaborative bridges between these isolated islands of expertise, thereby enhancing the network’s capacity for transformative, integrative innovation.

**Table 3:** Top 10 productive authors and their citation-normalized H-index indicators.

Number	Author	Count	WoS H-Index	Citation	H-Index/Citation
1	Yao, Juming [47]	10.0	61.0	2.0	30.5
2	MO, Liuting [48]	6.0	10.0	4.0	2.5
3	Shaojian, Lin [49]	6.0	32.0	2.0	16
4	Liu, Lin [50]	6.0	33.0	3.0	11
5	Gao, Junkuo [44]	6.0	62.0	4.0	15.5
6	Le, Phung [51]	5.0	23.0	2.0	11.5
7	Lan, Jianwu [52]	5.0	35.0	2.0	17.5
8	Zhang, Xiong-Fei [53]	5.0	41.0	0.0	N.A.
9	Jin, Chunde [54]	5.0	42.0	4.0	10.5
10	Ren, Penggang [55]	5.0	53.0	2.0	26.5

Note: Count and citation frequency were calculated from the 463-record corpus, whereas H-index was retrieved from WoS author profiles. H-index/Citation was used as an auxiliary citation-normalized indicator. N.A. indicates that the ratio was not calculated because the corpus citation frequency was zero.

**Table 4:** Top 10 most cited authors in the field.

Number	Cited Author	Count	Centrality	Year
1	LI J [45]	65	0.32	2015
2	LIU Y [46]	51	0.29	2017
3	ZHANG H [56]	42	0.09	2019
4	JIANG F [57]	41	0.19	2017
5	ZHANG Y [58]	39	0	2021
6	YANG J [59]	35	0.08	2022
7	WANG Y [60]	34	0.12	2020
8	LI Y [61]	33	0.04	2020
9	WAN CC [62]	31	0.02	2018
10	LI YQ [63]	29	0.05	2020

### 3.3. Knowledge Base and Theoretical Evolution

**Figure 7:**
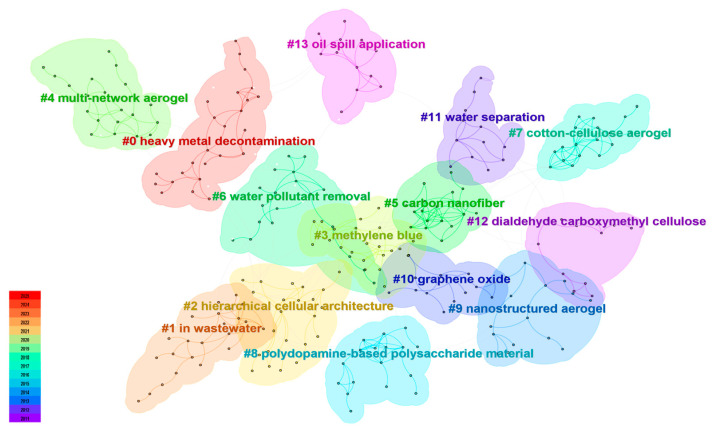
Knowledge structure and thematic clusters. Note: “#” denotes the CiteSpace cluster index.

To map the intellectual foundations of the field, a co-citation analysis was performed. The resulting network (282 nodes, 444 links) exhibits high modularity and coherence (Modularity Q = 0.7936; Mean Silhouette S = 0.9275), validating the robustness of the identified knowledge domains (Figure 7). The network resolves into 14 major clusters (Table 5), collectively revealing that the field is built upon two dominant functional axes: adsorptive sequestration and catalytic degradation. In this co-citation context, references with high co-citation counts represent frequently shared intellectual foundations, whereas references with high betweenness centrality act as bridges connecting different material-design routes and water-treatment themes.

The intellectual architecture is anchored by substantial clusters representing these dual foundations. Cluster #0 (heavy metal decontamination, Size = 25, S = 0.919) consolidates seminal work on physicochemical mechanisms (e.g., ion exchange) for pollutant capture, reflecting the field’s deep roots in adsorbent design and optimization [64,65]. Concurrently, clusters focused on catalytic processes (e.g., #3 methylene blue, #10 graphene oxide) aggregate foundational literature on integrating photocatalytic or Fenton-active components for pollutant mineralization via advanced oxidation processes (AOPs) [66,67,68]. The high betweenness centrality of references that pioneered advanced adsorbent design (e.g., Gao et al., 2018 in Table 6) underscores the foundational strength and critical role of the adsorption pillar [69]. These works provide the essential material platforms and enrichment capacity upon which the later integration of catalytic functions is built. The field’s defining paradigm—the intentional synergistic coupling of adsorption (for enrichment) and (photo)catalysis (for destruction)—is thus conceptually rooted in the complementary nature of these two pillars, and is operatively realized in later, convergent research that actively combines their principles, as seen in emerging clusters like #4 [70,71].

More specifically, the highly co-cited references listed in Table 6 contributed to water-treatment science by establishing several transferable design principles rather than merely reporting individual adsorption capacities. For oil/water separation and organic-solvent cleanup, representative studies clarified the importance of surface-wettability regulation, hydrophobic modification, capillary-driven uptake, compressibility, and recyclability in biomass-derived aerogel sorbents [69,72,73]. For heavy-metal and dye removal, bio-inspired polydopamine/PEI functionalization, amine-rich cellulose networks, chitosan-based interfaces, and clay- or MOF-containing composite aerogels demonstrated how chelation, ion exchange, electrostatic attraction, and enlarged active-site density can enable rapid and simultaneous removal of inorganic and organic pollutants [73,74,75,76,77]. In addition, studies on hierarchical, anisotropic, and shape-recoverable aerogels showed that pore-channel engineering and mechanical resilience are essential for improving mass transfer, operational stability, and cyclic reuse in wastewater-treatment scenarios [73,77,78]. Therefore, these highly co-cited works collectively transformed cellulose-based aerogels from simple porous sorbents into structurally engineered and chemically functionalized platforms for selective separation, multi-pollutant capture, regeneration, and subsequent integration with catalytic or photocatalytic degradation.

**Figure 8:**
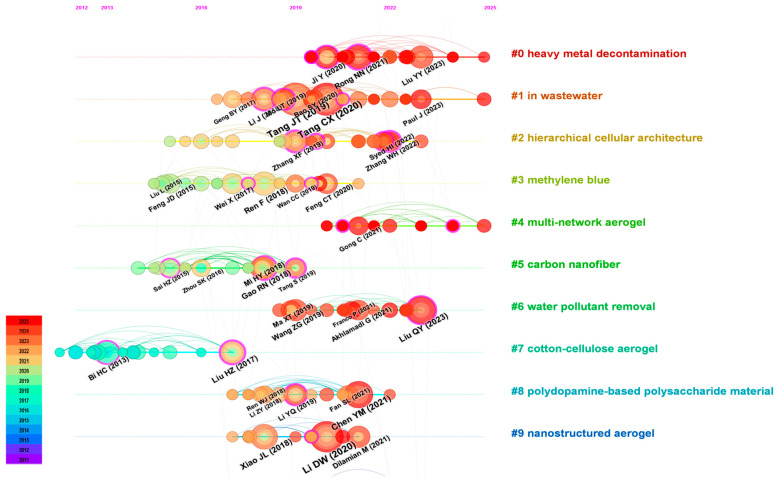
Temporal evolution of foundational knowledge clusters. Node labels (Author, Year) are auto-generated by CiteSpace to denote representative publications within each cluster. Note: “#” denotes the CiteSpace cluster index.

Temporally, the evolution of these knowledge clusters (Figure 8) demonstrates a clear trajectory from divergence to convergence. Early, parallel development in material synthesis and functional exploration gave way, post-2018, to a phase of intense cross-cluster interaction and synthesis. This convergence fueled the field’s exponential growth by integrating design principles from previously distinct tracks, including surface functionalization, hierarchical pore construction, wettability control, mechanical recovery, and adsorption-enabled pollutant enrichment. The most significant trend is the emergence of later clusters (e.g., #4 multi-network aerogel, Avg. year 2022) [71,79,80,81], which signify a mature phase of convergent synthesis. These clusters represent the active fusion of design principles from both foundational pillars, aiming to create integrated, multifunctional platforms for complex water-treatment applications, including multi-pollutant removal, regeneration, and adsorption-assisted catalytic or photocatalytic degradation. This evolution—from dual-track development to integrated design—encapsulates the field’s theoretical maturation.

**Table 6:** Highly co-cited foundational literature and their scientific contributions to water treatment.

Literature (First Author, Year)	Co-Citation Count	Centrality	Cluster ID	Title	Scientific Contribution to Water Treatment
Liu HZ, 2017 [72]	13	0.3	#7	Review on the Aerogel-Type Oil Sorbents Derived from Nanocellulose	Established nanocellulose aerogels as renewable sorbent platforms for oil/water separation by summarizing the roles of hydrophobic modification, high porosity, capillary uptake, compressibility, and recyclability in oil and organic-solvent removal.
Tang JT, 2019 [74]	27	0.01	#1	Compressible cellulose nanofibril (CNF) based aerogels produced via a bio-inspired strategy for heavy metal ion and dye removal	Demonstrated bio-inspired polydopamine/PEI functionalization as an effective interfacial-chemistry strategy for simultaneous heavy-metal and dye removal, highlighting chelation, electrostatic attraction, and multi-pollutant adsorption in compressible CNF aerogels.
Tang S, 2019 [78]	7	0.29	#5	Dye adsorption by self-recoverable, adjustable amphiphilic graphene aerogel	Clarified the importance of tunable wettability and elastic recovery for cyclic dye adsorption, showing that mechanically recoverable amphiphilic aerogels can maintain adsorption performance during repeated wastewater-treatment operation.
Li DW, 2020 [75]	26	0.08	#9	Multifunctional adsorbent based on metal-organic framework modified bacterial cellulose/chitosan composite aerogel for high efficient removal of heavy metal ion and organic pollutant	Introduced MOF-modified bacterial cellulose/chitosan aerogels as high-surface-area multifunctional adsorbents, demonstrating that MOF-derived active sites can broaden cellulose aerogels toward simultaneous metal-ion and organic-dye removal.
Tang CX, 2020 [73]	25	0.06	#1	Shape recoverable and mechanically robust cellulose aerogel beads for efficient removal of copper ions	Advanced cellulose aerogels from fragile monoliths toward shape-recoverable bead-type adsorbents, improving handling, structural stability, and rapid Cu(II) uptake for more practical water-treatment configurations.
Gao RN, 2018 [69]	13	0.24	#5	Mussel Adhesive-Inspired Design of Superhydrophobic Nanofibrillated Cellulose Aerogels for Oil/Water Separation	Established mussel-inspired surface modification as a route to convert hydrophilic nanofibrillated cellulose into superhydrophobic, oil-selective aerogels, contributing key principles for selective oil/water separation and reusable sorbent design.
Bi HC, 2013 [82]	12	0.1	#7	Carbon Fiber Aerogel Made from Raw Cotton: A Novel, Efficient and Recyclable Sorbent for Oils and Organic Solvents	Demonstrated that low-cost biomass fibers can be converted into recyclable carbon aerogels for oil and organic-solvent cleanup, providing an early scalable route for biomass-derived porous sorbents in water remediation.
Zhang XF, 2019 [76]	10	0.24	#2	Biohybrid Hydrogel and Aerogel from Self-Assembled Nanocellulose and Nanochitin as a High-Efficiency Adsorbent for Water Purification	Showed that crosslinker-free nanocellulose/nanochitin hybridization can create complementary surface functionalities for simultaneous inorganic and organic pollutant capture, linking green assembly with high-capacity water purification.
Rong NN, 2021 [77]	14	0.24	#0	Adsorption characteristics of directional cellulose nanofiber/chitosan/ montmorillonite aerogel as adsorbent for wastewater treatment	Demonstrated that directional freezing and clay/biopolymer hybridization can generate anisotropic channels and mechanically reinforced networks, improving mass transfer and stability during heavy-metal adsorption.
Chen YM, 2021 [83]	18	0.08	#8	Recent Progress on Nanocellulose Aerogels: Preparation, Modification, Composite Fabrication, Applications	Integrated preparation, modification, composite fabrication, and application knowledge for nanocellulose aerogels, providing a design framework for translating porous cellulose networks into multifunctional water-treatment materials.

Note: “#” denotes the CiteSpace cluster index; clusters are numbered in descending order of size, with #0 representing the largest cluster.

### 3.4. Research Hotspots and Frontier Evolution

Building upon the dual-pillared knowledge base, this section employs dynamic keyword analyses to map the current research agenda, revealing how foundational theories are translated into concentrated investigative fronts. Importantly, high-frequency keywords and burst terms are interpreted as indicators of shifting scholarly attention rather than direct evidence of technological maturity; therefore, the following analysis also evaluates whether these emerging themes represent substantive conceptual advances, incremental performance optimization, or still-unresolved translational challenges.

#### 3.4.1. Thematic Architecture: A Landscape of Catalytic Synergy

**Figure 9:**
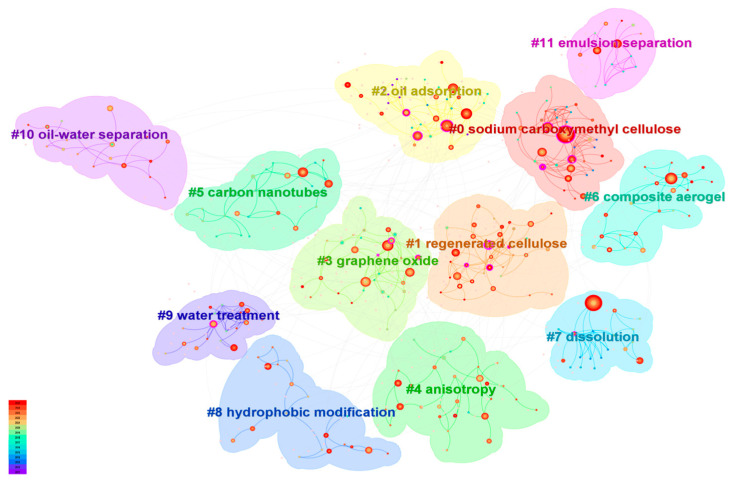
Keyword Co-occurrence Network. Note: “#” denotes the CiteSpace cluster index.

The keyword co-occurrence network (N = 314, E = 698), characterized by high modularity (Q = 0.7008) and internal coherence (S = 0.8654), resolves into 12 thematic clusters (Figure 9, Table 7). The research landscape is predominantly structured around the catalytic pillar. The largest cluster, #0 photocatalytic properties (Size = 38, S = 0.902) [24,83], acts as the central hub. Its high-frequency keywords—adsorption, degradation, and composites—directly articulate the core synergy of adsorption-enriched photocatalytic degradation [84,85,86]. This mechanism is further elaborated by Cluster #2 (photocatalytic degradation), which emphasizes fabrication and nanoparticles. Alongside these core mechanistic themes, mature application-oriented clusters such as #3 (oil spillage cleanup) [87,88] and #9 (water treatment) demonstrate a sustained focus on targeting specific pollutants [89,90,91]. The exceptionally high silhouette value of Cluster #7 (sewage treatment, S = 0.970) signifies a particularly well-defined and cohesive sub-field [64,92]. Simultaneously, the emergence of newer clusters like #6 (recyclable reduced graphene, Avg. year 2021) and #10 (ultralight dual network graphene aerogel, Avg. year 2021) [93] marks the current frontier in material innovation [94]. These clusters seek to enhance the aforementioned synergistic functionality through advanced nano-engineering, aiming for systems with greater efficiency and durability.

A more critical reading of the keyword structure, however, suggests that the field remains partly performance-driven and model-system-oriented. The frequent occurrence of terms such as methylene blue, Congo red, aqueous solution, efficient, performance, and adsorption performance indicates that many studies still evaluate cellulose-based aerogels using simplified pollutant systems and capacity- or efficiency-centered metrics. Similarly, the prominence of photocatalytic properties and photocatalytic degradation does not necessarily imply that fully integrated adsorption–catalysis processes have been achieved at the system level; in many cases, these keywords may reflect the incorporation of catalytic nanoparticles into aerogel matrices rather than validated continuous, regenerable, or real-wastewater treatment systems. Therefore, the thematic architecture should be understood as evidence of an ongoing transition from material functionalization toward process-relevant integration, rather than as proof that application-ready catalytic platforms have already been established.

#### 3.4.2. Temporal Trajectory: From Functional Demonstration to System Integration

**Figure 10:**
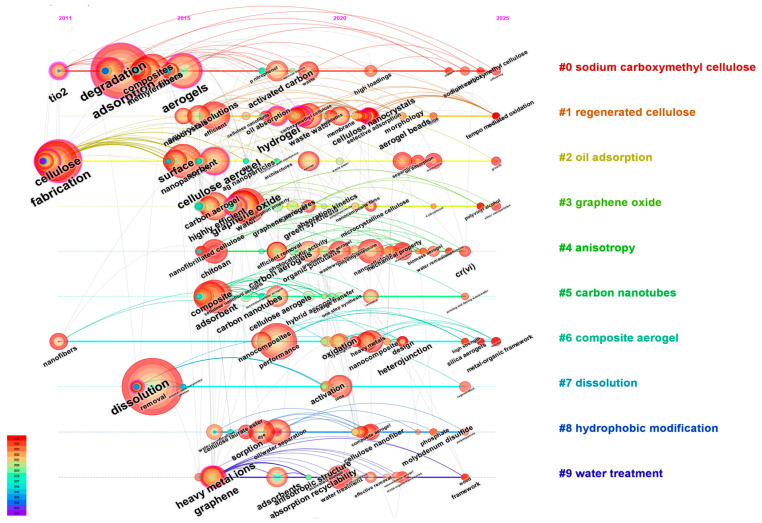
Keyword Timeline View. Note: “#” denotes the CiteSpace cluster index.

The keyword timeline view (Figure 10) delineates the field’s phased evolution, aligning with its logistic growth pattern. The Nascent Phase (circa 2011–2017) was characterized by foundational clusters centered on proof-of-concept for specific functions. Early-stage clusters such as #2 oil adsorption and #5 carbon nanotubes established the groundwork for pollutant capture and nano-material integration, respectively [95,96,97].

The subsequent Exponential Growth Phase (approx. 2018–2022) witnessed the vigorous expansion and interconnection of the synergistic core. The dominant cluster #0 sodium carboxymethyl cellulose [98], alongside specialized clusters like #3 graphene oxide (focusing on hybrid structures) and #8 hydrophobic modification (targeting oil-water separation) [99,100,101,102], drove intensive optimization of material performance for model pollutants. This period consolidated the adsorption-catalysis synergy as the central paradigm.

The ongoing Maturation Phase (post–2022) reveals a strategic evolution within this established framework. The sustained prominence of #9 water treatment and the emergence of integrative concepts such as #6 composite aerogel and #1 regenerated cellulose signal a critical transition [66,103,104]. Research focus is increasingly shifting from maximizing the performance of individual materials in idealized settings toward addressing system-level integration challenges, including scalability, process compatibility, recyclability, and functionality in complex aqueous matrices. Nevertheless, this transition should be interpreted as an emerging orientation rather than an accomplished transformation. The keyword evidence indicates that the field has begun to recognize the importance of regeneration, realistic water-treatment conditions, and integrated material architectures, but it does not yet demonstrate that these challenges have been systematically resolved. In this sense, the maturation phase is best characterized as a movement from “high-performance catalytic materials” toward “robust, application-ready catalytic systems,” with the latter still requiring stronger evidence from long-term cycling, continuous-flow operation, anti-fouling evaluation, and real-wastewater validation.

#### 3.4.3. Active Frontiers: Engineering Sustainable and Integrated Catalytic Platforms

**Figure 11:**
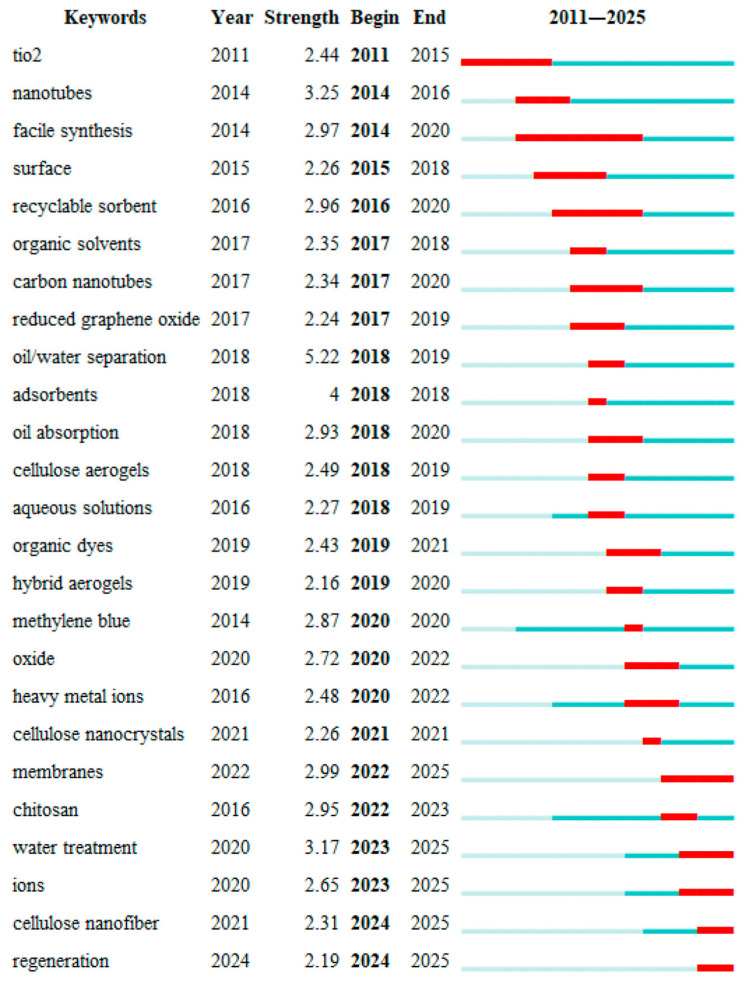
Keyword Citation Bursts.

Burst detection analysis (Figure 11) reveals that the current research frontier is characterized by a cohesive shift toward practical engineering applications of sustainable catalytic systems. While the strongest historical burst, oil/water separation (Strength = 5.22, 2018–2019), symbolized the peak of application-driven growth, recent and ongoing bursts through 2025 converge on several interconnected priorities.

Importantly, this frontier shift should be interpreted with appropriate caution. Keyword burst intensity captures changes in scholarly attention, but it does not necessarily indicate immediate technological maturity or field-readiness [105]. Burst detection can be influenced by corpus growth, keyword normalization, time slicing, and the delayed appearance of emerging terms in controlled vocabularies. In this dataset, the recent bursts in water treatment, membranes, regeneration, and cellulose nanofiber clearly signal the field’s movement toward application-oriented research; however, these signals should be read as indicators of research momentum rather than direct evidence that scale-up barriers have already been resolved. This distinction is essential because many frontier studies still rely on short-cycle batch tests, simplified pollutant solutions, and limited long-term validation.

A primary focus is system integration for practical application, as evidenced by sustained bursts in water treatment (2023–2025) and membranes (2022–2025). These keywords signal a move beyond batch-scale material testing toward the design of scalable, multifunctional systems that synergistically combine separation and degradation processes. This aligns with the broader vision of creating integrated, multi-step catalytic systems that operate efficiently out of thermodynamic equilibrium.

Concurrently, the theme of material circularity is prominent, highlighted by the burst keyword regeneration (2024–2025). This reflects a critical research drive to address catalyst longevity, recyclability, and overall economic viability, ensuring that advanced materials can transition from laboratory curiosities to industrially relevant technologies. Achieving this circularity necessitates the design of robust, reusable catalytic platforms. Complementing this is the emphasis on advanced and sustainable material precursors, exemplified by the burst of cellulose nanofiber (2024–2025). This focus underscores the ongoing refinement of biomass-derived nanomaterials to bolster the sustainability profile of the entire catalytic platform.

To improve translational credibility, future frontier studies should pair removal performance with deployment-relevant evidence. Priority should be given to regeneration durability over extended cycles, anti-fouling behavior in realistic ionic and organic backgrounds, stability under continuous or semi-continuous flow, and preliminary techno-economic or life-cycle indicators. Recent LCA/TEA studies on reactive filtration systems for wastewater treatment demonstrate that process feasibility depends not only on pollutant removal but also on material dose, energy demand, reagent consumption, service life, and recovery or disposal pathways [106]. Applying such a reporting framework to cellulose-based aerogel systems would help distinguish genuinely scalable advances from high-performing but condition-specific laboratory demonstrations.

Collectively, this frontier analysis confirms that the field’s cutting edge has evolved beyond the mere discovery of new photocatalytic materials. The research momentum is now firmly directed toward engineering these materials into durable, recyclable, and intelligently integrated components for practical, sustainable water remediation. This trajectory effectively fulfills the advanced objectives embedded in the original retrieval strategy, prioritizing regeneration, recycling, and process-level application.

## 4. Critical Scientific Discussion on Wastewater-Treatment Applications

### 4.1. Adsorption-Oriented Wastewater Treatment Functions

Adsorption remains the most mature and operationally robust function of cellulose-based aerogels in water remediation. The co-citation structure (Table 5), particularly clusters #0 (heavy metal decontamination), #3 (methylene blue), and #6 (water pollutant removal), indicates that pollutant capture has served as the primary technical entry point for this field. This prominence is scientifically reasonable: adsorption provides rapid contaminant enrichment, low auxiliary energy demand, and broad applicability to metal ions and dye molecules across diverse concentration windows. Recent records identified through the adsorption-oriented validation step further illustrate the breadth of adsorption applications, including dye sorption and oil-water separation in textile wastewater treatment, phosphorus capture from real domestic wastewater, benchmarking of dye-pollutant adsorbents, molybdenum ion adsorption and metal recovery, and organic dye adsorption by MOF/cellulose aerogel composites [107,108,109,110,111].

From a structure–function perspective, adsorption performance is governed by the interplay of hierarchical porosity, interfacial chemistry, and wetting behavior. Open porous frameworks facilitate mass transport, while functional groups on cellulose-derived backbones provide binding sites for electrostatic attraction, coordination, hydrogen bonding, and π-related interactions, depending on pollutant chemistry. Representative adsorbent-oriented systems (e.g., hydrophobic carbon aerogel variants) further demonstrate that interface design can be tuned for different classes of contaminants, including oils and organics. Recent non-China-led studies further confirm that adsorption performance is strongly controlled by pore accessibility, surface functional group density, and interfacial polarity matching between adsorbent and pollutant, especially for mixed dye/metal systems under realistic ionic backgrounds [35,37,112].

However, adsorption-dominant routes also show intrinsic limits. First, high apparent removal may partly reflect phase transfer rather than true detoxification. Second, competitive adsorption in multi-solute matrices often reduces selectivity and capacity. Third, repeated sorption–desorption cycles can induce structural fatigue, active-site loss, or incomplete regeneration. These limitations explain why the field is progressively moving from “adsorption as endpoint” toward adsorption-enabled integrated treatment architectures. Consistent with broader international evidence, adsorption-only systems are increasingly evaluated as front-end concentration units rather than complete detoxification solutions, motivating coupling with catalytic or oxidative polishing steps [36].

Representative studies consistently report strong wastewater-treatment performance of cellulose-based aerogels across dye, heavy-metal, and oil-containing systems, while highlighting matrix dependence. In batch tests, high removal efficiencies are commonly achieved for model dyes and metal ions due to combined porosity-enabled transport and surface-site interactions. Functionalized systems (e.g., amine-rich, polydopamine-assisted, or conductive hybrid frameworks) further improve uptake kinetics and selectivity, particularly in mixed contaminant conditions. For oil/water scenarios, hydrophobic or amphiphilic aerogel designs generally show rapid sorption and effective phase separation. However, performance attenuation during cycling and under complex ionic backgrounds remains recurrent, confirming that “high initial removal” and “long-term deployability” are not equivalent endpoints. Therefore, reported results should be interpreted jointly with regeneration stability, structural retention, and process-mode validation (batch vs. flow-through), rather than by single-cycle peak removal alone.

### 4.2. Catalytic/AOP-Related Pathways

Catalytic integration addresses a central weakness of purely adsorptive systems by enabling pollutant transformation rather than only capture. In this context, cellulose-based aerogels have evolved into multifunctional scaffolds for hosting photoactive or oxidant-activating components. Early demonstrations of enhanced photocatalytic behavior in polymer-decorated cellulose aerogels (Ref. 39) established the feasibility of coupling adsorption-mediated enrichment with in situ degradation. Later work on peroxymonosulfate (PMS) activation in recyclable MOF/cellulose aerogels (Ref. 43) further expanded this strategy into advanced oxidation process (AOP) regimes. Recent international reports (2022–2025) show that cellulose-derived porous supports can improve catalyst dispersion, interfacial charge transfer, and oxidant utilization efficiency in photocatalytic and PMS/PDS-assisted AOP configurations [113,114,115].

Mechanistically, two complementary pathways are now evident. The first relies on photoinduced charge generation and interfacial redox reactions, where the aerogel matrix improves pollutant accessibility to catalytic domains. The second employs oxidant activation (e.g., PMS) to generate reactive species (or non-radical oxidative routes), enabling rapid decomposition of recalcitrant organics. In both cases, cellulose-derived porous networks are not merely passive supports; they regulate diffusion fields, local concentration gradients, and catalyst dispersion states.

Despite clear progress, catalytic/AOP systems still face practical constraints, including activity decay during cycling, catalyst leaching risk, inhibition by background ions/natural organic matter, and uncertainty in byproduct toxicity. Scientifically, these limitations indicate that performance is controlled not only by intrinsic catalyst activity, but also by mesoscale transport fields, interfacial microenvironment, and support–active phase coupling stability. Therefore, future studies should adopt a mechanism-grounded evaluation framework that links (i) reaction pathways and oxidant utilization, (ii) structural/chemical stability of the aerogel scaffold, and (iii) effluent safety endpoints (mineralization and toxicity proxies). This evidence chain is essential to avoid over-claiming based on apparent removal alone and to establish robust criteria for practical wastewater deployment [112].

### 4.3. From Single-Function Systems to Integrated Processes

A major conceptual transition in this field is the shift from single-function materials to process-integrated treatment systems. This transition is consistent with the temporal signals in the knowledge structure and hotspot evolution, where recent clusters emphasize multifunctionality, composite design, and application-facing integration. In practical terms, the objective is no longer to maximize one metric (e.g., adsorption capacity or initial degradation rate) in isolation, but to engineer coordinated workflows that combine capture, conversion, and recovery. This process-integration trend is aligned with recent water-treatment literature advocating hybrid treatment trains in which adsorption, catalytic oxidation, and separation modules are co-designed for stability under variable water matrices [116].

Three integration patterns are emerging. First, intra-material integration combines adsorptive and catalytic functions within a single aerogel architecture, enabling sequential enrichment–degradation in one unit. Second, module-level integration embeds aerogel components into membranes or flow-through reactors to improve operational controllability. Third, process-level integration links pretreatment, catalytic polishing, and regeneration steps into closed or semi-closed loops. Across these patterns, design logic is converging toward stable performance in complex water matrices rather than peak performance in idealized batch tests.

Accordingly, evaluation criteria should be updated. Alongside removal efficiency, studies should systematically report continuous-flow stability, throughput-normalized performance, resistance to fouling/inhibition, regeneration efficiency, and function retention over multiple cycles. This metric shift is essential for translating laboratory innovation into credible engineering options. Accordingly, internationally proposed evaluation frameworks now prioritize flow-mode robustness, fouling tolerance, and throughput-normalized performance over single-point batch maxima.

### 4.4. Practicality: Regeneration, Recyclability, and Deployment Barriers

Regeneration and recyclability are now decisive benchmarks for technological relevance, consistent with the retrieval emphasis on regenerat* and recycl*. In mature application scenarios, a high-performing material without reliable regeneration is unlikely to be economically or environmentally competitive. For cellulose-based aerogel systems, the key challenge is to regenerate function while preserving structural integrity and interfacial activity.

Recent progress has diversified the regeneration and reuse strategies for cellulose-based aerogels. For metal-ion adsorption systems, pH-regulated elution, salt-assisted desorption, and chelating-agent washing are commonly used to release bound ions and recover active sites. For dye- and organic-contaminant removal, solvent washing, surfactant-assisted desorption, and polarity-adjusted regeneration have been explored to overcome strong electrostatic, hydrogen-bonding, or π-related interactions. For oil/water separation, thermal, mechanical squeezing, and photothermal regeneration have been reported to restore sorption capacity while maintaining hydrophobic interfaces. In catalytic or photocatalytic aerogels, reaction-assisted self-cleaning has become particularly attractive because adsorbed pollutants can be degraded in situ by photoinduced oxidation, PMS/PDS activation, Fenton-like reactions, or other AOP pathways, thereby converting aerogels from single-use sorbents into recyclable separation–reaction platforms [117,118].

Nevertheless, regeneration remains one of the main bottlenecks for engineering deployment. Many studies still report only a limited number of cycles, use simplified regeneration media, or evaluate recyclability mainly through residual removal efficiency. Such evidence is insufficient to confirm long-term reusability. Repeated regeneration may induce mass loss, swelling/shrinkage, pore collapse, mechanical fatigue, surface-functional-group depletion, catalyst leaching, and changes in selectivity under complex water matrices. For adsorption–catalysis integrated aerogels, failure pathways are even more complicated because adsorption sites, catalytic centers, and the cellulose skeleton may degrade at different rates. Therefore, regeneration should be evaluated not only by short-term recovery ratios but also by capacity retention, cumulative treated-water volume, mass-balance closure, structural diagnostics, catalyst leaching, regeneration chemical demand, energy input, and secondary-waste generation over extended cycles.

Beyond material-level issues, deployment barriers are increasingly system-level: scale-up reproducibility, batch-to-batch consistency, compatibility with real wastewater variability, and the absence of harmonized testing protocols across studies. To close the lab-to-field gap, future work should adopt three parallel practices: (i) standardized benchmarking under realistic water chemistries, (ii) continuous or semi-continuous reactor validation, and (iii) joint techno-economic and life-cycle assessments. Such a framework would allow the field to progress from “promising materials” to “deployable treatment technologies” with transparent performance claims and credible sustainability evidence. From a translation perspective, coupling harmonized performance protocols with techno-economic assessment (TEA) and life-cycle assessment (LCA) is increasingly recognized as a minimum requirement for judging deployment readiness of emerging adsorbent–catalyst materials [88,119].

From an environmental and economic feasibility perspective, cellulose-based aerogels have clear advantages but also non-negligible constraints for large-scale water treatment. Their main advantages arise from renewable feedstocks, low density, high porosity, and the possibility of integrating adsorption, separation, and catalytic functions within one material platform. These features may reduce material consumption and enable compact treatment units. However, large-scale feasibility will depend strongly on the full production and operation chain rather than on removal efficiency alone. Energy-intensive drying processes, solvent exchange, chemical crosslinking or hydrophobic modification, incorporation of costly nanocatalysts, secondary chemical consumption during regeneration, and end-of-life management may offset part of the environmental benefit of cellulose as a renewable precursor. Economically, key determinants include raw-material availability, fabrication throughput, drying energy, catalyst or functional-agent cost, service lifetime, regeneration frequency, and the achievable treated-water volume per unit mass of aerogel. Therefore, future LCA/TEA studies should compare cellulose-based aerogel systems with established adsorbents, membranes, and catalytic treatment units under equivalent influent quality, treatment targets, and operating modes.

## 5. Conclusions, Limitations, and Future Perspectives

### 5.1. Key Findings

Within the predefined wastewater-treatment and water-purification scope, this work delivers a bibliometric and visual review of cellulose-based aerogels based on 463 publications indexed in the Web of Science Core Collection from 2011 to 2025. The field exhibits a clear S-shaped growth trajectory, evolving from nascent exploration to rapid expansion and then to a maturation stage marked by consolidation rather than mere volume increase.

At the social-network level, research activity is strongly concentrated in East Asia, with China contributing the largest publication share in the present dataset. Meanwhile, participation from multiple countries and regions has continued to expand, indicating a broader international research landscape. However, institutional and author-level networks remain relatively fragmented, resembling an “archipelago of micro-teams.” This structure has supported rapid parallel innovation, but it also limits cross-group knowledge integration and may constrain system-level breakthroughs.

At the intellectual level, the field has progressed from parallel development to functional convergence. Early advances were largely distributed across two dominant functional axes—adsorptive sequestration and catalytic degradation—whereas current hotspots increasingly emphasize their integration into enrichment–destruction workflows. Frontier topics now extend beyond maximizing single-material performance toward engineering robust, recyclable, and application-oriented treatment systems.

Overall, the main scientific finding of this review is that cellulose-based aerogels are no longer being developed only as high-capacity porous adsorbents; rather, they are increasingly being designed as multifunctional platforms that integrate adsorption, separation, catalytic degradation, regeneration, and process compatibility. This shift has important implications for future research and practical application. Scientifically, it suggests that material design should focus on coupled structure–function relationships, including hierarchical mass transport, interfacial binding chemistry, catalyst–support stability, and regeneration-induced structural evolution. Practically, it indicates that deployment readiness should be judged not by single-cycle peak removal alone, but by long-term functional retention, real-wastewater robustness, continuous-flow performance, environmental footprint, and economic feasibility.

### 5.2. Study Limitations

Several limitations should be acknowledged when interpreting the present bibliometric and visualized findings. First, the dataset is restricted to English-language articles and reviews indexed in the Web of Science Core Collection. Although WoSCC was selected because of its curated metadata, standardized cited-reference records, and suitability for CiteSpace-based science mapping, this database choice inevitably constrains literature coverage. Relevant studies indexed only in other databases, reported in non-English literature, published as conference papers, patents, dissertations, technical reports, or not captured by the selected search query may therefore be underrepresented. Consequently, country-level, institution-level, and author-level publication shares should be interpreted as characteristics of the retrieved WoSCC corpus rather than absolute measures of global research output. Relatedly, the quantitative maps represent corpus-based patterns rather than exhaustive counts of all cellulose-based aerogel studies for wastewater treatment. Records identified only through the adsorption-oriented validation search were used for qualitative contextualization and were not merged into the predefined network dataset [120].

Second, bibliometric indicators and visualized networks are sensitive to data-cleaning decisions and software parameter settings. Citation-based indicators may be affected by citation delay, field-size effects, self-citation, database coverage, and differences in publication age. Network structures generated by bibliometric visualization tools such as CiteSpace depend on time slicing, threshold selection, g-index settings, pruning algorithms, clustering resolution, and keyword normalization. Cluster labels generated by log-likelihood ratio or related algorithms provide useful thematic cues, but they may oversimplify heterogeneous research topics. Co-citation, co-authorship, and keyword co-occurrence networks reveal statistical associations rather than causal relationships, and burst signals indicate rapid growth in scholarly attention rather than direct evidence of technological maturity. Therefore, the bibliometric results in this study should be interpreted as structured evidence of research evolution and emerging attention, and should be complemented by expert assessment, standardized experimental benchmarking, real-wastewater validation, continuous-flow testing, and integrated TEA/LCA evaluation.

### 5.3. Future Perspectives

Future progress should prioritize translation from high-performing materials to deployable treatment technologies. A first priority is sustainable fabrication. Although cellulose provides a renewable starting point, the overall sustainability of cellulose-based aerogels depends on the complete production route, including solvent use, crosslinking chemistry, drying method, functional-agent loading, and waste management. Future studies should therefore move toward water-based or solvent-minimized processing, greener crosslinkers, lower-energy drying technologies, ambient-pressure drying when feasible, direct use of low-cost biomass or industrial cellulose residues, and reduced dependence on scarce or expensive catalytic additives. Sustainable design should also consider mechanical durability, shape retention, and regeneration compatibility at the material-design stage, rather than treating them as post-synthesis performance tests.

A second priority is industrial implementation. Research should move beyond small monolithic aerogel samples and batch adsorption tests toward manufacturable and process-compatible formats, including beads, granules, membranes, coated supports, cartridges, and flow-through modules. Pilot-scale studies should evaluate hydraulic stability, pressure drop, fouling resistance, mass-transfer limitations, regeneration logistics, catalyst or functional-agent leaching, treated-water throughput, and compatibility with existing wastewater-treatment trains. Standardized performance protocols are also needed, including continuous-flow operation, multi-cycle regeneration, real-wastewater matrices, cumulative treated-water volume, and effluent-safety endpoints such as mineralization and toxicity proxies.

Finally, regeneration, recyclability, environmental impact, and cost should be assessed together rather than separately. Combined TEA/LCA frameworks are needed to compare cellulose-based aerogel systems with established adsorbents, membranes, and catalytic treatment units under equivalent influent quality, treatment targets, and operating modes. Given the currently fragmented collaboration pattern, structured interdisciplinary consortia are also needed to establish shared benchmarks, interoperable datasets, cross-laboratory validation pipelines, and pilot demonstrations. Such coordinated efforts are essential for narrowing the gap between laboratory innovation and scalable water-remediation practice.

## Figures and Tables

**Table 1:** Top 10 countries in terms of publication count.

Number	Country	Count	Year of First Publication	Centrality
1	PEOPLES R CHINA	347	2014	1.07
2	CANADA	19	2016	0.01
3	USA	17	2017	0.18
4	IRAN	16	2018	0.07
5	INDIA	15	2020	0.05
6	SOUTH KOREA	15	2018	0.01
7	VIETNAM	12	2019	0.02
8	JAPAN	11	2017	0.02
9	EGYPT	9	2021	0.07
10	MALAYSIA	8	2015	0.06

**Table 2:** Top 15 institutions in terms of publication numbers.

Number	Institution	Count	Year of First Publication	Centrality	Cumulative Percentage (%)
1	Nanjing Forestry University	22	2017	0.1	6.8
2	Sichuan University	17	2018	0.15	12.1
3	Zhejiang Sci-Tech University	15	2016	0.02	16.8
4	Donghua University	13	2021	0	20.8
5	Chinese Academy of Sciences	13	2017	0.17	24.8
6	Guangxi University	11	2022	0.12	28.3
7	Jiangsu University	10	2018	0	31.4
8	Shaanxi University of Science & Technology	10	2021	0.02	34.5
9	South China University of Technology	8	2021	0	37.0
10	Northeast Forestry University—China	8	2017	0.01	39.4
11	Beijing Forestry University	8	2021	0	41.9
12	Dalian Polytechnic University	8	2020	0.14	44.4
13	Zhejiang A&F University	7	2016	0.02	46.6
14	Egyptian Knowledge Bank (EKB)	7	2021	0	48.8
15	Vietnam National University Ho Chi Minh City (VNUHCM) System	6	2022	0	50.6

**Table 5:** Characteristics of major co-citation clusters.

Cluster ID	Size	Silhouette	Label (LLR)	Average Year
#0	25	0.919	heavy metal decontamination	2021
#1	24	0.962	in wastewater	2020
#2	24	0.896	hierarchical cellular architecture	2019
#3	23	0.864	methylene blue	2017
#4	20	0.953	multi-network aerogel	2022
#5	19	0.872	carbon nanofiber	2016
#6	19	0.957	water pollutant removal	2021
#7	18	0.984	cotton-cellulose aerogel	2013
#8	17	0.978	polydopamine-based polysaccharide material	2019
#9	15	0.929	nanostructured aerogel	2019
#10	14	0.979	graphene oxide	2019
#11	13	0.833	water separation	2019
#12	12	0.897	dialdehyde carboxymethyl cellulose	2019
#13	12	0.974	oil spill application	2021

Note: “#” denotes the CiteSpace-generated cluster index; clusters are numbered in descending order of size, with #0 representing the largest cluster.

**Table 7:** Characteristics of major keyword co-occurrence clusters.

Cluster ID	Size	Silhouette	Label (LLR)	Representative Keywords (Top 5 by Frequency)	Average Year
#0	38	0.902	photocatalytic properties	adsorption (124), aerogels (54), methylene blue (52), degradation (38), composites (31)	2016
#1	36	0.771	composite microsphere	efficient (41), wastewater (25), aqueous solutions (23), hydrogel (19), carboxymethyl cellulose (18)	2020
#2	36	0.942	photocatalytic degradation	fabrication (78), cellulose (66), nanoparticles (51), cellulose aerogel (40), absorption (25)	2014
#3	33	0.865	oil spillage cleanup	water (55), graphene oxide (44), aqueous solution (43), highly efficient (31), carbon aerogel (21)	2018
#4	30	0.801	recent trend	chitosan (25), nanocellulose (23), carbon aerogels (17), efficient removal (17), organic pollutants (16)	2020
#5	24	0.802	stable cellulose-based porous binary metal–organic gel	adsorbent (48), composite (33), cellulose aerogels (21), bacterial cellulose (15), cu(ii) (12)	2018
#6	22	0.888	recyclable reduced graphene	performance (55), nanocomposite (22), nanocomposites (18), heavy metals (17), nanofibers (12)	2021
#7	20	0.97	sewage treatment	removal (135), ions (24), dissolution (7), nanotubes (5), activation (4)	2016
#8	20	0.786	Congo red	oil/water separation (28), dye (27), water purification (10), cellulose nanofiber (9), composite aerogel (8)	2020
#9	19	0.915	water treatment	graphene (25), heavy metal ions (21), oil (21), water treatment (20), adsorbents (17)	2019
#10	19	0.838	ultralight dual network graphene aerogel	oxide (13), recyclable sorbent (13), fiber (12), robust (6), adsorption performance (6)	2021
#11	14	0.954	blue dye	aerogel (47), photocatalytic degradation (31), dye adsorption (17), reduction (10), TiO_2_ nanoparticles (3)	2019

Note: “#” denotes the CiteSpace cluster index; clusters are numbered in descending order of size, with #0 representing the largest cluster.

## Data Availability

The data presented in this study are available on request from the corresponding author. The data are not publicly available due to privacy restrictions.

## Supplementary Materials

The following supporting information can be downloaded at: https://www.mdpi.com/article/10.3390/gels12070643/s1, Supplementary File S1: Search strategy, screening workflow, and adsorption-oriented validation analysis; Supplementary File S2: Complete list of the 463 publications included in the bibliometric corpus.
