# Supplementary material for: A Bibliometric and Critical Review of Cellulose-Based Aerogels for Wastewater Treatment
Source: Gels. 2026 Jul 18;12(7):643. doi: 10.3390/gels12070643 (PMC13409539; doi:10.3390/gels12070643)
Supplement: Supplementary file 1 [file gels-12-00643-s001.zip › Supplementary Files/Supplementary File S1. Search strategy, screening workflow, and adsorption-oriented validation analysis.docx]

**Search strategy, screening workflow, and adsorption-oriented validation analysis**

**S1.1.** **Database source and retrieval settings**

The bibliometric dataset was retrieved from the Web of Science Core Collection. The search was conducted using predefined field tags and was limited according to the time window, language, and document-type constraints described below. All records were exported with full records and cited references for bibliometric analysis.

**Database:** Web of Science Core Collection

**Search date:** 14 January 2026

**Time span:** 2011–2025

**Language:** English

**Document types:** Article and Review

**Export format:** Full Record and Cited References

**Final corpus size:** 463 records

**S1.2. Main search strategy**

Adsorption-related terms were not used as mandatory search terms in the main query. Instead, the predefined corpus was constructed to capture cellulose-based aerogel studies within a wastewater/water-treatment context while allowing for multiple treatment or performance dimensions, including adsorption-related removal, catalytic/photocatalytic degradation, material regeneration, recyclability, separation, and integrated treatment pathways. Because adsorption-specific descriptors were not mandatory in the main query, an adsorption-oriented validation analysis was conducted after finalizing the corpus.

The main query used in the Web of Science Core Collection was as follows: TS=((cellulose OR nanocellulose OR "microcrystalline cellulose" OR MCC OR CNF) AND aerogel* AND (wastewater OR "water treatment" OR "water purification" OR dye* OR pollutant* OR contaminant*) AND (photo-Fenton OR photocataly* OR "advanced oxidation process" OR regenerat* OR recycl*)).

**Table S1. Stepwise retrieval and screening workflow for the predefined bibliometric corpus.**

| **Step** | **Operation** | **Search field/filter** | **Records retained** | **Notes** |
| --- | --- | --- | --- | --- |
| 1 | Initial retrieval using the predefined main query | WoS Core Collection; field tags as specified in the query | 520 | Initial topic-field retrieval |
| 2 | Time-span restriction | 2011-2025 | 469 | Same time window used throughout the study |
| 3 | Language restriction | English | 467 | Non-English records excluded |
| 4 | Document-type restriction | Article and Review | 466 | Non-target document types excluded |
| 5 | Deduplication | WoS export and manual check | 463 | Duplicate records removed |
| 6 | Eligibility confirmation by title/abstract screening | Inclusion/exclusion criteria listed in Table S2 | 463 | No additional records were excluded after deduplication; this step confirmed the final predefined bibliometric corpus. |

**S1.3. Inclusion and exclusion criteria**

Records were screened according to predefined inclusion and exclusion criteria. The aim was to retain publications focusing on cellulose-based aerogels in wastewater or water-treatment contexts.

**Table S2. Inclusion and exclusion criteria used for title/abstract screening.**

| **Category** | **Criteria** |
| --- | --- |
| Inclusion criterion 1 | The study involved cellulose, nanocellulose, cellulose nanofiber, cellulose nanocrystal, bacterial cellulose, microcrystalline cellulose, carboxymethyl cellulose, or other cellulose-derived components. |
| Inclusion criterion 2 | The material was described as an aerogel, cryogel, porous aerogel-like monolith, or cellulose-based aerogel composite. |
| Inclusion criterion 3 | The application was related to wastewater treatment, water purification, pollutant removal, dye removal, heavy-metal removal, oil/water separation, catalytic degradation, photocatalysis, or other water-remediation functions. |
| Inclusion criterion 4 | The publication was a research article or review within the predefined document-type and language constraints. |
| Exclusion criterion 1 | The study focused on cellulose materials but not aerogels or aerogel-like porous monoliths. |
| Exclusion criterion 2 | The study focused on aerogels but not cellulose-based or cellulose-derived materials. |
| Exclusion criterion 3 | The study was unrelated to wastewater treatment, water purification, or environmental remediation. |
| Exclusion criterion 4 | The record was a correction, editorial, meeting abstract, book chapter, news item, or other non-target document type, if outside the predefined document-type constraints. |
| Exclusion criterion 5 | Duplicate or incomplete records were removed before analysis. |

**S1.4. Adsorption-oriented validation analysis**

Because adsorption is a major treatment function of cellulose-based aerogels, an adsorption-oriented validation analysis was conducted after finalizing the predefined 463-record corpus. This validation analysis was designed to evaluate the representation of adsorption-focused studies without reconstructing the original bibliometric corpus.

Two internal validation searches were first conducted within the predefined corpus. The following adsorption-related descriptors were used: adsorp* OR adsorbent* OR sorp* OR sequestrat*. The wildcard terms were selected to capture common variants such as adsorption, adsorptive, adsorbent, adsorbed, sorption, sorbent, and sequestration. The internal validation searches were: #1 AND TS=(adsorp* OR adsorbent* OR sorp* OR sequestrat*) and #1 AND TI=(adsorp* OR adsorbent* OR sorp* OR sequestrat*), where #1 denotes the predefined 463-record corpus.

A targeted title-level validation search was then performed to identify highly relevant adsorption-wastewater studies that were outside the predefined corpus. To maintain high specificity, the targeted validation search required adsorption-related descriptors to co-occur in the article title with explicit cellulose-based aerogel descriptors and wastewater/water-treatment descriptors.

The targeted validation search was: TI=(("cellulose aerogel*" OR "cellulose-based aerogel*" OR "nanocellulose aerogel*" OR "microcrystalline cellulose aerogel*" OR "bacterial cellulose aerogel*" OR "cellulose nanofiber aerogel*" OR "cellulose nanofibre aerogel*" OR "cellulose nanocrystal aerogel*" OR "carboxymethyl cellulose aerogel*" OR "wood-derived cellulose aerogel*" OR "CNF aerogel*" OR "MCC aerogel*") AND (adsorp* OR adsorbent* OR sorp* OR sequestrat*) AND (wastewater OR "wastewater treatment" OR "water treatment" OR "water purification")). The same database, time window, language, and document-type constraints as the main search were applied.

**Table S3. Adsorption-oriented validation searches.**

| **Search ID** | **Query** | **Purpose** | **Records** |
| --- | --- | --- | --- |
| #1 | Predefined main corpus | Main bibliometric dataset | 463 |
| #A | #1 AND TS=(adsorp* OR adsorbent* OR sorp* OR sequestrat*) | Topic-level adsorption coverage within the predefined corpus | 342 |
| #B | #1 AND TI=(adsorp* OR adsorbent* OR sorp* OR sequestrat*) | Title-level adsorption coverage within the predefined corpus | 140 |
| #C | TI=(("cellulose aerogel*" OR "cellulose-based aerogel*" OR "nanocellulose aerogel*" OR "microcrystalline cellulose aerogel*" OR "bacterial cellulose aerogel*" OR "cellulose nanofiber aerogel*" OR "cellulose nanofibre aerogel*" OR "cellulose nanocrystal aerogel*" OR "carboxymethyl cellulose aerogel*" OR "wood-derived cellulose aerogel*" OR "CNF aerogel*" OR "MCC aerogel*") AND (adsorp* OR adsorbent* OR sorp* OR sequestrat*) AND (wastewater OR "wastewater treatment" OR "water treatment" OR "water purification")) | Targeted title-level adsorption-wastewater validation search | 8 |
| #C NOT #1 | Records retrieved by #C but absent from #1 | Additional adsorption-focused records outside the predefined corpus | 5 |

**S1.5. Additional records identified by the targeted adsorption-oriented validation search**

The targeted title-level validation search identified five additional records that were not included in the predefined 463-record corpus. These records were not merged into the quantitative bibliometric network dataset because the bibliometric analysis was based on a predefined and reproducible corpus. Instead, they were considered in the qualitative discussion of adsorption mechanisms and wastewater-treatment applications.

**Table S4. Additional records identified outside the predefined corpus through the adsorption-oriented validation search.**

| **No.** | **Article title** | **Application context** | **Main treatment function** | **Use in the revised manuscript** |
| --- | --- | --- | --- | --- |
| 1 | Cellulose-based aerogels for efficient dye sorption and oil-water separation in textile wastewater treatment | Textile wastewater; dye removal; oil-water separation | Sorption and separation | Considered in the qualitative discussion of adsorption-oriented applications |
| 2 | Synergistic adsorption of real phosphorus-containing domestic wastewater by in-situ growth of MgFe-layered double hydroxides co-doped with dual-functional lignosulfonate and La(OH)3 on wood-derived cellulose aerogel | Domestic wastewater; phosphorus removal | Adsorption | Considered in the qualitative discussion of adsorption-oriented applications |
| 3 | Nanostructured Cellulose-Based Aerogels: Influence of Chemical/Mechanical Cascade Processes on Quality Index for Benchmarking Dye Pollutant Adsorbents in Wastewater Treatment | Dye-contaminated wastewater | Adsorption and performance benchmarking | Considered in the qualitative discussion of adsorption-oriented applications |
| 4 | Advancing wastewater treatment and metal recovery: Aminated ZIF-8 composite cellulose aerogel as an innovative biomass adsorbent for enhanced molybdenum ion adsorption | Wastewater treatment; molybdenum ion removal; metal recovery | Adsorption and resource recovery | Considered in the qualitative discussion of adsorption-oriented applications |
| 5 | Adsorbent based on MOF-5/cellulose aerogel composite for adsorption of organic dyes from wastewater | Organic dye removal from wastewater | Adsorption | Considered in the qualitative discussion of adsorption-oriented applications |

**S1.6. Treatment of validation records in the bibliometric analysis**

The quantitative bibliometric analyses, including co-citation analysis, keyword analysis, burst detection, and collaboration network mapping, were conducted using the predefined 463-record corpus. The additional five records identified through the adsorption-oriented validation search were used only to support the qualitative discussion of adsorption-related wastewater-treatment functions.

This treatment was adopted to maintain methodological consistency and reproducibility of the quantitative network analysis while ensuring that recent and highly relevant adsorption-focused studies were considered in the critical discussion.
